# Development and Psychometric Validation of the Music Receptivity Scale

**DOI:** 10.3389/fpsyg.2020.585891

**Published:** 2021-01-08

**Authors:** Mahesh George, Judu Ilavarasu

**Affiliations:** Division of Yoga and Physical Sciences, Swami Vivekananda Yoga Anusandhana Samsthana, Bengaluru, India

**Keywords:** musical identity, music perception, factor analysis, psychometrics, music receptivity

## Abstract

A new construct, termed music receptivity, is introduced and discussed in this work. Music receptivity can be defined as a measure of the extent of internalization that an individual has, to a given piece of music, as measured at the point of listening. Through three studies, we demonstrate the psychometric properties of the construct—the Music Receptivity Scale (MRS). Exploratory factor analysis on a sample of 313 revealed good psychometric validity, with a four-factor solution (emotional experience, interest, attention, and hurdles), with a Cronbach’s alpha of 0.89, and a two-factor solution (emotion experience and attention), with a Cronbach’s alpha of 0.87. The tool also had a good test–retest reliability (*r* = 0.87 for a 15 day interval and *r* = 0.91 for 1 month interval). Overall, the tool had 20 items in the long form and 12 items in the short version. The MRS could distinguish musicians and non-musicians supporting its discriminant validity. We have also discussed the implication of the MRS in the field of music psychology.

## Introduction

Musical applications have been burgeoning in recent years. Music has been extensively used in different fields like psychotherapy (Metzner, 2004; Grocke et al., 2008; Lin et al., 2011), education (Larsson and Georgii-Hemming, 2019), and sports (Belkhir et al., 2019). Understanding how music influences the human mind and how it induces and modulates mood states has been always a key question that researchers are trying to address. Existing research has focused on using music in therapy (Brown and Jellison, 2012; Chu et al., 2014), finding neural correlates of music (Saari et al., 2018), music perception (Koelsch, 2011), etc. Subjective human experiences to music are an area which is broad and complex (Cowen et al., 2020). Therefore, understanding individual differences in music listening is of colossal significance. Investigating how individuals experience music can give a profound insight on how music influences the human mind. There are a few tools like the Absorption in Music Scale (Sandstrom and Russo, 2013) and the Music Involvement Scale (Nagy and Szabó, 2004), which attempt to measure the degree of absorption and involvement in music. However, they assess more of a trait construct. Hence, there is a need for a tool that can assess the extent or depth of internalization of music, in a given context of listening along with the stable traits of the person. Internalization can mean the extent to which the given piece of music is taken in or absorbed by an individual listening to it, leading to a feeling of deeply resonating with the music. Hence, there remains a need to develop a suitable tool assessing the degree of internalization. To fill this lacuna, we propose a construct, music receptivity, and a psychometric tool—the Music Receptivity Scale (MRS)—to measure the same. Music receptivity is a measure of the extent or depth of internalization that an individual has, to a given piece of music, as measured at the point of listening. This tool could appraise the nature and intensity of subjective human experiences in music listening. Music receptivity can be correlated with several other constructs in music psychology. In the following sections, we present the concept music receptivity, the factors influencing it, and its psychometric validation.

### Need for a New Construct of Music Receptivity

The search for traits that uniquely identify a person’s inclination toward music has been a curious quest (Schäfer, 2016). Miranda (2020) identified that neuroticism is associated with musical habits like the extent of music listening, musical sensibility (emotional reactions), music preferences (music genres), and functions of music (emotion regulation). Miranda et al. (2010) likewise found that, in high school students, extraversion and openness alone are related to music preference and not the other dimensions of the big five personality factors. A meta-analysis reported a small to medium correlation between neuroticism and emotion regulation through music listening (Miranda and Blais-Rochette, 2018). Even though these studies successfully hint toward deep-rooted psychological traits associated with musical abilities, we need a construct that shows how these traits dynamically interact and result in different musical experiences such as elation, thrills, chills, feeling moved, and awestruck. The construct music receptivity might justify this requirement.

Music perception restricts to basic perceptual aspects of music, like pitch, rhythm, tempo, etc., and predominantly, the cognitive processes associated with it (Koelsch, 2011). These physical attributes are well studied and elucidated to show how they invoke a higher-order experience (Justus and Bharucha, 2002; Deutsch, 2007). Levitin et al. (2018) have discussed temporal factors in music as a preliminary step in understanding how and why music literally moves us. However, in order for music to have a transformational influence and reconfigure psychological states, it must be accompanied by higher-order cognitive and emotional processing, which may lead to one experiencing higher-order mind–body experiences (Juslin and Västfjäll, 2008; Sandstrom and Russo, 2013). For music therapy to be effective, this higher dimension of processing has to occur in the individual. The emphasis in this study is toward internal psychological processes. Unlike music perception, where different physical features can have identifiable thresholds and ranges, we propose music receptivity to follow a psychological continuum varying from a lower degree to a higher degree of receptivity. This differential receptivity would have a direct implication in standardizing music for therapy in various clinical conditions. An individual-centric approach to music therapy is needed, as music listening is a highly subjective phenomenon (Schäfer et al., 2013). Therefore, it is important to know how and to what extent did a piece of music affect an individual while he listened to it. This motivated us to think of the construct—music receptivity. Music perception is about the ability of an individual to perceive or distinguish the parameters of a given piece of music such as pitch, rhythm, and tempo, whereas music receptivity measures the extent of internalization that an individual may have to any given piece of music as measured at the point of listening. We can define internalization as the extent to which the given piece of music is taken in or absorbed by an individual listening to it, leading to a feeling of deeply resonating with the music. This is closer to the definition of absorption as defined by Tellegen and Atkinson (1974), as the willingness to be drawn in deeply, without distraction. This has been also associated with hypnotic susceptibility (Sandstrom and Russo, 2013). Unlike absorption that portrays the willingness to be drawn in, the internalization in music receptivity conveys to what extent the given piece of music has actually been deeply absorbed. An individual may have a higher degree of willingness to be absorbed with a musical piece, but due to other situation factors, the individual may have a different degree of music receptivity. The Music Receptivity Scale, which we have developed, gives two outcomes—the level of internalization (music receptivity) to a given piece of music and the nature of an individual’s subjective experience while listening to it. The music receptivity score can be graded as low, average, or high, and an individual’s subjective experiences may be assessed by the first item of the Music Receptivity Scale, which is the “emotions/feelings table.” These factors strongly determine, at the time of listening to a piece of music, how much that piece is internalized by the person. Semantics and affect could be the important means through which music can invoke higher-order experiences, along with the interaction of situational attention and a preset interest. Hence, this construct was proposed to have the qualities of both trait as well as state. Music receptivity, therefore, can enable us to understand various related concepts and theories in music psychology.

The process of evoking emotional responses while listening to music is a complex phenomenon. From the framework of music receptivity, any response to listening to music can be considered as a combination of two sets of processes—internal (dispositional) and external (situational). The domains of the MRS, i.e., emotion, interest, lyrical appraisal, attention, and hurdles, were proposed attributing trait characteristics. This means we expect that any individual will have trait emotional response patterns, trait interest, trait lyrical appraisal, trait attention, and trait hurdle (given their nature of personality). However, these components may not always be expressed in a predictable manner in different contexts. Hence, we have emphasized that music receptivity is specifically looked into when there is an interaction happening between these trait components and the situational factors like nature of music, ambiance of listening, emergent situational factors, affective, and cognitive state of the person, etc. So, finally, MRS can be considered as a combination of trait and state aspects, but with a greater weightage on the trait aspects. That is the reason, developing a scale, instead of a checklist, to measure music receptivity is meaningful. So, the major domains of the MRS are theoretical in nature and a few situational factors, external factors, which could be encompassed as hurdles, are an atheoretical construct.

### Music Receptivity: Definition

Music receptivity is defined as the measure of the extent of internalization that an individual has, to a given piece of music, while listening to it, as measured at the point of listening. Music receptivity constitutes five domains. They are attention, interest, lyrical appraisal, emotional experience, and hurdles.

**FIGURE 1:**
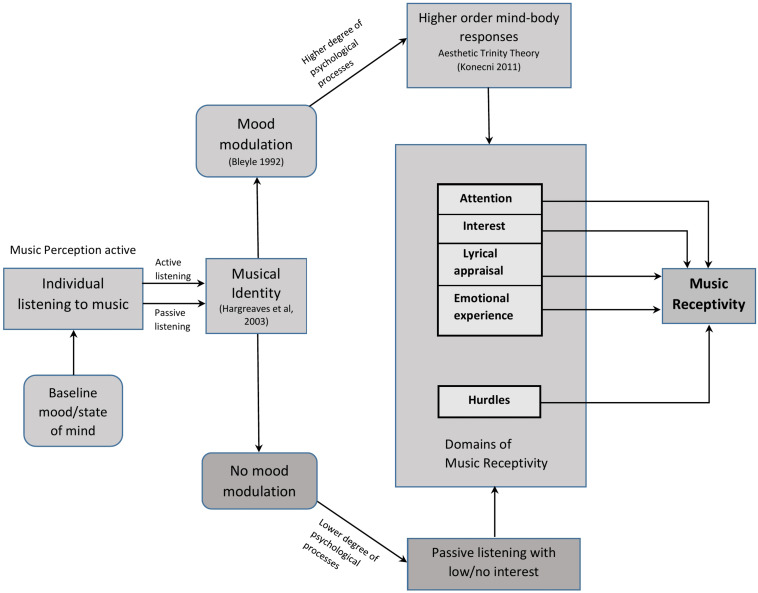
Conceptual framework of music receptivity.

Figure 1 shows the conceptual diagram of the musical receptivity construct. We propose that the major domains of music receptivity are attention, interest, lyrical appraisal, emotional experience, and hurdles.

(1) Attention: The ability of an individual to focus efficiently to a given musical piece, at the point of listening, in the presence or absence of external/internal disturbances or hurdles.
(2) Interest: This comprises both state and trait interest. State interest is when someone listens to a music piece and finds it novel in one or the other way or when develops a sudden liking for any reason. This is similar to situational interest. Situational interest is elicited by aspects of an object or a situation, such as novelty, intensity, or by interest-inducing factors, contributing to the attractiveness of the situation (Tobias, 1994; Krapp, 1999). Trait interest is very similar to the idea of musical identity. The development of people’s musical identities begins with biological predispositions toward musicality and is then shaped by the people, groups, situations, and social institutions that they encounter as they develop in a particular culture (Hargreaves and Marshall, 2003). Music receptivity of an individual to any given piece of music may be strongly mediated by the individual’s musical identity.
(3) Lyrical appraisal: The extent to which an individual understands and appreciates the lyrical content in a given piece of music.
(4) Emotional experience: The sum total of all the feelings and emotions evoked through the cognitive and affective processes occurring in an individual while he listens to a given piece of music. Juslin and Västfjäll (2008) proposed a theoretical framework featuring six psychological mechanisms of emotion induction through music—(1) brainstem reflexes, (2) evaluative conditioning, (3) emotional contagion, (4) visual imagery, (5) episodic memory, and (6) musical expectancy. They suggest that these mechanisms, along with cognitive appraisal, can explain most emotions induced by music in everyday life.
(5) Hurdles: Hurdles is the fifth domain which captures any barriers related to ambiance, postures, etc. It can be internal and/or external distractions or difficulties that one may perceive while listening to a given piece of music. Internal distraction can be mental distraction and/or bodily distraction. Mental distractions can be due to intrapersonal communication, mind-wandering, etc. One may start mind-wandering for various reasons—due to lack of interest in the music being played, due to lack of focus and attention, or while listening to a song, a word or a line in the song might catch the fancy of an individual and push him to a daydreaming session. Bodily distractions while listening to music can occur due to any physical discomfort. External distraction are essentially physical distractions, e.g., sound of a passing vehicle, interrupting the individual while listening to music, etc.

### How Is Music Internalized?

We can assume a baseline mood state of mind just before an individual starts to listen to a given piece of music. This mood state may or may not change while listening to music. However, music is a powerful medium that can transform the individual’s current mood state to another mood state or enhance the existing mood state. This is called mood modulation (Bleyle, 1992). We can assume that the more the piece of music is in synchrony with the individual’s musical identity, the greater are the chances of mood modulation to happen. Music perception would happen when a person is listening to music, be it active or passive listening. However, we assume that music perception will be more with active listening than in passive listening. Figure 1 shows two pathways: top and bottom. Processes occurring through the top pathway may lead to high music receptivity, and the ones happening through the bottom pathway may result in average or low music receptivity. We propose a scheme of these pathways as below.

(1) Top pathway: When an individual is listening to a piece of music and at a point of time if the music transitions to correspond to his musical identity, his interest and attention increases, emotional experience increases, and lyrical appraisal increases if he can appreciate the lyrics. Mood modulation happens as the music continues to correspond to his musical identity. Emotional experience is always high when mood modulation happens. When mood modulation happens, a higher degree of psychological processes may occur and higher-order mind–body responses (comprises physiological chills, thrills, tears, etc., feeling moved, esthetic awe) (Konečni, 2011) may be exhibited. All of these lead to a high music receptivity. Depending on to what extent the piece of music is in synchrony with the individual’s musical identity, the extent of music receptivity increases. Music receptivity is mediated by the musical identity of the individual listening. Hurdles, be it internal or external, may be overcome to a certain extent if the music is in synchrony with the musical identity of the individual. Beyond a certain extent, it reduces music receptivity.
(2) Bottom pathway: When a person listens to music, and the music does not correspond to his musical identity, mood modulation does not occur and this results in a lower degree of psychological processes. What ensues is passive listening with low/no interest. These may lead to average or low music receptivity. Attention, interest, lyrical appraisal, and emotional experience may all be low or average in this case. In the bottom pathway, the chances of hurdles to affect the individual’s listening are high as the individual may already be distracted.

Transition may happen from the bottom pathway to the top pathway or vice versa based on variations in the musical identity of the individual.

### Other Tools That Attempt to Assess Related Constructs

The Music Self-Concept Inventory (MSCI) was developed to evaluate change or development in music self-concept, and it has three subscales: support or recognition from others, personal interest or desire: and self-perception of music ability (Hash, 2017). The Interpersonal Music-Communication Competence Scale was developed to measure a set of abilities that can help develop the interpersonal communication through music training (Hald et al., 2017). The Music Perception Ability Questionnaire was developed to assess the general perception ability of non-musicians and enable them to assess their music abilities (Law and Zentner, 2012). The Music USE (MUSE) questionnaire was developed to assess the engagement styles of eight different background music and provide an overall qualitative and quantitative measure of music use. This questionnaire had shown four distinct engagement styles: cognitive and emotion regulation, engaged production, social connection, and dance and physical exercise (Chin and Rickard, 2012). The Motivation for Learning Music (MLM) questionnaire, based on the self-determination theory (Deci and Ryan, 1985), was developed to assess the degree of inherent motivation of music students to learn music (Comeau et al., 2019). The Adaptive Functions of Music Listening Scale was another novel perspective to assess various music listening functions related to general well-being. This questionnaire has 11 domains like stress regulation, anxiety regulation, anger regulation, loneliness regulation, rumination, reminiscence, strong emotional experiences, awe and appreciation, cognitive regulation, identity, and sleep. Their study showed higher functions of music listening in females (Groarke and Hogan, 2018). To evaluate the influence of home ambiance of music exposure and engagement in infants and preschool children, the Music@Home questionnaire was developed, and this study also revealed distinct patterns of parents’ music characteristics (Politimou et al., 2018). Based on the music model of motivation (Jones, 2009), the Music Model of Academic Inventory was developed in a music education setting. This tool had five domains, namely empowerment, usefulness, success, interest, and caring (Jones and Skaggs, 2016). A modular tool for music research to assess musicianship, musical capacity, music preferences, and motivations for music use was developed (Chin et al., 2018). The Barcelona Music Reward Questionnaire (BMRQ) was developed to assess the variations in how listeners experience reward in any music-related activities (Mas-Herrero et al., 2013). The Goldsmiths Musical Sophistication Index (Gold-MSI) measures inherent characteristics like musical skill, expertise, achievements, and other such related behaviors related to a variety of musical contexts. This tool gives an estimate of mastery of a person in a particular area of music and can reflect musical talent, ability, aptitude, or musical potential (Müllensiefen et al., 2014). Observing these studies, a basic need to develop a tool that helps in understanding the core internal processes that clearly define how a person responds to music is revealed. The uniqueness of the Music Receptivity Scale is in its ability to accommodate situational as well as inherent factors to determine the extent of internalization to a given piece of music.

A psychometric tool that could measure music receptivity and at the same time appraise the nature and intensity of subjective experiences in music listening could have immense applications in a varied set of contexts. For a clinical setup, a smartphone-based application could be developed, where the MRS would serve as an integral part. This application could help manage client databases to music therapy, continually evaluate client responses to tailor-made music interventions, assess musical identities of clients, etc. Most of all, the MRS would act as a feedback tool for the music therapist. Individuals could download such an application to their smartphones and self-evaluate their responses to a particular piece of music that they listened to. Such an application would play a significant role in everyday music listening in the lives of the average man. Having discussed potential applications and the relationship of this novel construct to various theories in music psychology, we now present the psychometric development and validation of the Music Receptivity Scale.

## Materials and Methods

The research design was a mixed design, using both qualitative and quantitative methods. The research study was approved by the Institutional Ethics Committee, and the details are given in the OSF link: https://osf.io/v8jb9/.

### Phase 1—Qualitative Study

Toward refining the construct and generating items for the psychometric instrument, we conducted seven in-depth personal interviews and also a focus group discussion involving seven subject matter experts from a leading music college in Kerala, a state in south India. The average duration of these interviews was 1 h. The inclusion criteria were as follows: post-graduates, Ph.D. holders in music with an expertise of 10 years and above in the field of music education and research, expert performers/exponents of music, and willing to participate (informed consent was taken from each of these experts). For in-depth interviews, we had musicians with an experience of performing/composing and/or teaching and research experience of over 10 years (*n* = 7; Professor-1, Associate Professor-1, Assistant Professor-1, Music composers-2, Performers-2). For the focus group discussion (FGD), we had seven experts (Assistant Professors-4, Associate Professor-1, Professors-2) from the same department of music. The medium of the interview was Malayalam, the local language of Kerala. Interviews were conducted until data became redundant. The interviewees’ comments, views, and suggestions were extracted while transcribing the audio recording of the interviews. The interviews were manually transcribed and coded. The interviews were unstructured and questions were asked based on eight probe questions (see OSF link: https://osf.io/v8jb9/ for details). Following data analysis, we generated items and consolidated the questionnaire with 23 items (details on the items are given in the OSF link: https://osf.io/v8jb9/). Following this, two experts of psychometrics were also consulted and their advice was sought toward the overall refinement of the tool.

### Phase 2—Quantitative Study

For the quantitative study, the research design employed was survey design and the sample size was calculated using the rule of thumb (Nunnally, 1978), which states that the subject to item ratio for exploratory factor analysis should be at least 10:1, i.e., 10 individuals for each item. We took an excess of 14 individuals per item for the field validation study (*n* = 322). The convenience sampling technique was employed.

#### Content Validity

Once the final form of the tool (23 items) was consolidated after the qualitative study, content validation documents were prepared [three documents were handed over to each of the 12 experts—(1) about the study: (2) the Music Receptivity Scale, the actual handout which would be used for the field study;, and (3) content validation sheet] and we met with each of the 12 experts. Similar inclusion criteria were used as in the case of the qualitative study, and the experts were briefed in person about the study. The three documents were handed out to them and they returned the content validation sheets after validating in due time. Lawshe’s content validation ratio was calculated for each item, and as per the literature, for a number of 12 experts, the content validity ratio (CVR) should be 0.56 and above (Lawshe, 1975). Three items (4, 16, 21) had CVR below 0.56. We retained all three items for the field validation study. The rationale why they were retained is as follows: Item 4: “*I was distracted due to daydreaming while listening to the given music.*” was similar to item 12: “*While listening to the given music, I was losing focus, going back and forth on daydreaming*,” which had high CVR and hence we retained it to add more items to the domain. Item 16 was: “*I associated disturbing/unpleasant memories or events with this piece of music.*” This item has its importance in a clinical music therapy setting. Individuals may associate disturbing memories or traumatic events that happened in their life to the music that they might have heard during that phase of life. Listening to such unpleasant and disturbing music might trigger memories yielding to negative clinical effects. Therefore, item 16 was retained to identify such responses. Item 21 was: “*While listening to the music, I was imaginative/creative.*” This item taps the creative dimension of music listening, which we consider an important aspect of music receptivity. Listening to music with high interest creates a unique imagery, which can help to identify musical identity. Active music listening is a creative activity in that the listener constructs a uniquely personal music experience (Kratus, 2017). All items were agreed upon by the experts to be both culturally relevant and easily comprehendible.

#### Face Validity

Once the content validation process was completed, we consolidated the tool and gave it out to 15 laymen in order to assess its face validity, and their responses ensured good face validity of the tool.

#### Field Testing of the Instrument

A pilot study was conducted (*n* = 63; 28 males, 35 females), involving all undergraduate engineering students (age range: 18–23 years). The study was conducted in a classroom setup employing floor-standing tower stereo speakers. The music we chose for the study is a popular song among the audiences of Kerala, a state in the south of India. The title of the song is “*Samayamithapoorva sayahnam*,” its duration was 5 min and 12 s, and this song is from the Malayalam language movie “*Harikrishnans*” (1998), composed by Ouseppachan, written by Kaithapram Damodaran Namboothiri (Fazil, 1998). This piece of music adheres to the Carnatic music/South Indian Classical Music tradition. The same song was used for the pilot study, field testing, and with the musicians. It is challenging to claim that this musical piece would evoke a homogeneous response in the target audience, as the listener’s characteristics may considerably vary. However, the rationale for choosing this song is given below:

(1) It is a semiclassical piece of music considered to have melodic content, and the major percussion instrument—“*mridangam*”*—*is intelligently used. We consider that these characteristics of the musical piece would create an uplifting experience for the listeners. The song is a *ragamaalika* (*Ragamaalika* is a term given to a music composition where more than one *ragas* are intelligently knit together) comprising of the *ragas*—*Navarasa kanada*, *Begada*, and *Desh*. All these ragas are known to induce feelings of love, compassion, devotion, and tranquility.
(2) It appeared to have clear, meaningful, and poetic lyrics (the song lyrics have been translated and given in the OSF link: https://osf.io/v8jb9/).
(3) The song and the lyrics together are considered to evoke the emotions/feelings of devotion, surrender, love, presence of the divine, pacifying/soothing, happiness, reflections on one’s life, etc.
(4) Overall, the song is such that any individual in general (in an Indian context) could appreciate it, and the chances of this piece of music evoking any negative feeling/emotion are considered to be minimal, according to our contention.

*Ragas* are modal melodies comprising the canon of North Indian classical music. Each raga is constructed from five or more musical notes, organized into one ascending sequence and one descending sequence of notes, which together comprise a single melodic framework. The performance of a raga is restricted within the note sequences of its ascending and descending halves, but is improvised in all other respects—e.g., timing between notes and sustain and attack of each note (Valla et al., 2017). The same definition of raga applies for the South Indian classical music as well.

The participants were asked to be seated comfortably and then briefed about the study, particularly the importance of this study in fostering field applications of music therapy in clinical settings. Then, the handouts (comprising the participant informed consent form, checklist of demographic information, checklist to screen for relevant disabilities/disorders, the 23-item MRS) were given out to all 63 participants present there. Once they filled out this information, they were instructed to not look into the handout further and keep it closed. Then, the music was played, and once the song ended, they were instructed to open the handout and the initial instruction was read out and explained to them. The initial instruction of the MRS is: “There are 35 emotions/feelings listed in these CELLS below. Please go through each of them; You may have EXPERIENCED many numbers of emotions/feelings given below, while you listened to the given music; go on, identify all those and rate them on a scale of 1–5 (score 1 as the lowest level of experience; score 5 as the highest level). Give your rating within the brackets. Please do not think much, your immediate response will be the best.” These instructions are for the first item of the MRS, which is validated separately from the rest of the 22 items. The 23-item tool used for the field validation study is given in the OSF link: https://osf.io/v8jb9/. There were three test administrators, and the participants were told that they could put up their hands and ask any questions if they had related to taking the test. Once they finished taking the test, the handouts were collected from them and data was entered into Excel sheet and prepared for analysis. The pilot data was subjected to analysis, and the psychometric properties of the tool were observed following which we decided to retain all 23 items for the field study.

The field validation study was conducted with a sample size of 313 (*n* = 313; 133 males; 179 females; 1 transgender). Data were taken from two colleges, both located in Trivandrum, a district in Kerala. The participants’ age range was from 18 to 22. The study was conducted in the amenity center of the college, which is designed to facilitate audio-visual entertainment and conducting college cultural programs. The auditorium had an excellent speaker system, and before the actual study was conducted, three test administrators played the music there in the auditorium and checked for the sound quality of the speakers there and ensured that the echo and reverberation was ideal to the piece of music selected. Then, the participants were brought to the auditorium and the same process was repeated similar to the pilot study, with the only difference that the initial instructions were elaborated more and they were briefed in detail about the difference between “knowing that there is a particular emotion/feeling present in a piece of music” and “experiencing a particular emotion/feeling within themselves while listening to a piece of music.” The second set of data was taken under similar conditions.

In order to evaluate the performance of the MRS on a sample trained in music, we selected students from a music college (males = 40, females = 4). In the context of our study, a musician can be defined as a person who practices any one or more instruments or vocal and has musical skills that people around him acknowledge. However, he need not be a music professional. In the music college, students engage with at least one of the musical instruments or with vocal practice. We anticipated that this group would show higher scores on music receptivity compared with the non-musicians.

## Results

### Data Screening and Extraction

Three sets of data, pilot (*n* = 63), general population (*n* = 313), musicians (*n* = 44), were separately analyzed. The data were initially extracted to an Excel and checked for any typo using the double entry method (Barchard and Verenikina, 2013) and screened for any possible outliers. It led to the removal of nine cases where there were clear indications of inappropriate entry or too many missing values. The final sample size used to report the results is 313, which is in the ratio of 13 cases per item, more than the recommended 10 cases per item. All the analyses were done using R statistical software, version 3.4.2 (R Development Core Team, 2020), and its psych package (Revelle, 2019).

### Qualitative Analysis of Interviews

#### In-Depth Interviews and Focus Group Discussion

To refine the construct of music receptivity and to get inputs for item generation, we conducted unstructured in-depth interviews and focus group discussion.

Some of the ideas that emerged from the in-depth interviews were as follows: the need to standardize the music and its duration appropriate to the clinical condition of an individual, the need to identify confounding factors in clinical application of music therapy, societal and cultural bases of emotion induction through music, precision of delivery of music, instrumental music may be therapeutically superior to other modes, and necessity to account for a person’s internal state while assessing the degree of music receptivity (excerpts of the in-depth interview are provided in the OSF link: https://osf.io/v8jb9/ for more information).

Some of the key ideas that came up from the FGD were as follows: the need to match the music intervention to the mental state of the participant, the need to assess musical preferences beforehand in music therapy, music sense is a differential ability, pure instrumental music could invoke sublime emotions, choice of music should be based on the personality and the preference of the person, perfection in music performance is necessary to induce strong emotions in listeners, layman may not understand the finer nuances of a piece of music yet still have an ability to appreciate music, importance of knowing any negative associations that one may have to certain types of music or a particular piece of music, trained musicians may prefer music over lyrics, innate interest greatly influences attention toward music, lyrics may not be necessary to bring out emotional experiences, etc. Furthermore, the experts equivocally opined that such an instrument which could measure the depth of internalization to music and also appraise the nature and intensity of subjective experiences in music listening was absolutely necessary (see OSF link: https://osf.io/v8jb9/).

#### Item Generation

Items were generated based on the inputs obtained from the in-depth interviews and the FGD. One of the authors of this article, who is a post-graduate in Applied Psychology, trained as an Indian classical vocalist with over 15 years of performance experience, and also an audiophile, prepared the items of the Music Receptivity Scale. Two psychometric experts from the Department of Psychology, University of Kerala were also consulted. The consolidated questionnaire had 23 items designed to capture the domains of attention, interest, lyrical appraisal, emotional experience, and hurdles. The first item had 35 items to capture the various emotions experienced and had the rating scale of 1–5, least to maximum experienced level of emotion. All the other 22 items had responses on a five-point Likert scale, strongly agree to strongly disagree. They were coded in such a way that a higher score indicated higher music receptivity.

### Factor Analysis

As the aim of this study was to evaluate the structure of the MRS, we used exploratory factor analysis to determine the number and nature of underlying factors of the MRS. We used parallel analysis to determine the number of factors to retain (Horn, 1965); principal axis factoring was performed to evaluate the number of underlying factors by employing oblique rotation (oblimin) as the domains were anticipated to be correlated. Maximum iterations for convergence were fixed at 1,000. The analyses revealed more than one solution. We report two possible solutions and another two-factor model with reduced items. We based our theoretical construct as the prime factor to decide upon the factor structure and then to check if it is further supported by the empirical data.

#### Factor Analysis of Pilot Data (*n* = 63)

Exploratory factor analysis on a sample of 63 was performed. A parallel analysis revealed two factors to be extracted. Bartlett’s test of sphericity was performed to check the suitability of performing factor analysis, and the result was statistically significant showing that factor analysis can be performed. The Kaiser–Meyer–Olkin (KMO) test, a measure of sampling adequacy, revealed that values for all the items were greater than 0.75 except for item numbers 3 (0.53), 16 (0.43), and 18 (0.62). The mean sampling adequacy was 0.84. Minimum value expected is 0.50, and 0.60 is mostly recommended. Pilot study data were used to optimize field administration and deduce adequate sample size and probable structure of the music receptivity construct. It was observed that the ambiance of the study setup including the sound system needed improvement.

#### Factor Analysis of the Main Data (*n* = 313)

A parallel analysis suggested three factors to be extracted. Overall mean sample adequacy (MSA) was 0.88. The MSA for individual items ranged from 0.40 to 0.94. The MSA for item 18 alone was lower (0.40) and all the other items’ MSA values were above 0.70. Bartlett’s test of sphericity was significant [χ^2^(231) = 2,468.79, *p* < 0.001]. Principal axis factoring was performed on all the 22 items to determine the underlying factors by employing oblique rotation (oblimin) as we anticipated that the factors will be intercorrelated. We, however, started by extracting five factors as per our theoretical predictions and later tried our four-factor solution. The four-factor solution was closer to our theoretical model. Also, the two items, item 3 (*I was comfortable with my posture while listening to the given music*) and item 18 (*The music played was loud for my ears*), were removed as they were weakly loaded. We accommodated these two items as part of the instruction statement. It was obvious that we must take care of external environmental conditions before we attempt to measure music receptivity. Principal axis factoring was again performed on the reduced 20 items. Overall MSA was 0.89, and for individual items, it ranged from 0.74 to 0.94. Bartlett’s test of sphericity was also significant [χ^2^(190) = 2,397.69, *p* < 0.001]. Table 1 shows the four-factor and two-factor solutions for the MRS.

**TABLE 1:** Exploratory factor analysis showing the factor loadings from the pattern matrix for the 20-item MRS scale (*n* = 313), with four-factor and two-factor solutions.

Items		Four-factor solution					Two-factor solution		
		Emotion	Interest	Attention	Hurdle	Communality	Affect	Attention	Communality
I17	The music “moved me”/“touched my heart.”	0.84				0.73	0.83		0.68
I23	The lyrics of the music “moved me”/“touched my heart.”	0.77				0.61	0.77		0.58
I15	The music took me to another world.	0.67				0.58	0.77		0.58
I11	The music brought back good memories.	0.62				0.5	0.67		0.45
I21	While listening to the music, I was imaginative/creative.	0.62				0.42	0.64		0.41
I8	I got emotionally triggered while listening to the given music.	0.54				0.39	0.6		0.38
I19	The music evoked images and/or connected thoughts in my mind.	0.4				0.3	0.55		0.3
I20	I understood the meaning of the lyrics well.	0.33				0.16	0.35		0.12
I14*	I did not like the lyrics of the given music.	0.31				0.31	0.42		0.27
I13	I would love to listen to this music again.	0.44	0.5			0.69	0.79		0.65
I2*	The given music was not interesting to me.		0.54			0.57	0.68		0.52
I5*	The given music sounded boring to me.		0.77			0.71	0.66		0.53
I4*	I was distracted due to daydreaming while listening to the given music.		0.35			0.26		0.49	0.24
I7*	My intensity of focus was varying while listening to the given music.			0.55		0.42		0.5	0.35
I22*	While listening to the music, I was disturbed/distracted by external factors.			0.57		0.3		0.4	0.16
I6*	It was difficult for me to be attentive while I was listening to the given music.		0.36	0.45		0.41		0.53	0.37
I9*	Although I wanted to be attentive on the whole, my attention was not up to the mark.			0.63		0.43		0.56	0.31
I16*	I associated disturbing/unpleasant memories or events with this music.				0.7	0.43		0.39	0.16
I10*	Disturbing thoughts came into my mind while listening to the given music.				0.58	0.5		0.64	0.4
I12*	While listening to the given music, I was losing focus, going back and forth on daydreaming.		0.38		0.31	0.48		0.68	0.45
	Cumulative variance	0.2	0.31	0.4	0.46		0.27	0.39	

*Items marked with ^∗^ are reverse coded. Loadings less than 0.30 are suppressed.*

The total variance explained by this four-factor 20-item scale is 46%.

Upon careful observation, we also noticed that some of the factors in the four-factor model are intercorrelated and they can be converged to a two-factor solution. Table 1 also depicts the two-factor solution. The cumulative variance for this 20-item two-factor solution is 39%.

We further refined the two-factor model by removing certain items that had relatively weaker loadings or that had considerable cross-loadings. For this, items 3, 4, 6, 12, 13, 14, 16, 18, 19, and 20 from the 22-item MRS were deleted. The resultant 12-item scale (Table 2) had two factors with total variance explained of 45%.

**TABLE 2:** Exploratory factor analysis showing the factor loadings from the pattern matrix for the 12-item short version of the MRS scale (*n* = 313), with two-factor solution.

	Items	Two-factor solution		
		Affect	Attention	Communality
I17	The music “moved me”/“touched my heart.”	0.84		0.72
I23	The lyrics of the music “moved me”/“touched my heart.”	0.79		0.62
I15	The music took me to another world.	0.76		0.57
I2*	The given music was not interesting to me.	0.68		0.49
I11	The music brought back good memories.	0.64		0.44
I5	The given music sounded boring to me.	0.63		0.44
I21	While listening to the music, I was imaginative/creative.	0.62		0.38
I8	I got emotionally triggered while listening to the given music.	0.61		0.4
I9*	Although I wanted to be attentive on the whole, my attention was not up to the mark.		0.66	0.44
I7*	My intensity of focus was varying while listening to the given music.		0.57	0.42
I10*	Disturbing thoughts came into my mind while listening to the given music.		0.53	0.28
I22*	While listening to the music, I was disturbed/distracted by external factors.		0.49	0.24
	Cumulative variance	0.33	0.45	

Items marked with * are reverse coded. Loadings less than 0.30 are suppressed.

We propose to label the two-factor solution as affect and attention. Under affect, both emotion and interest, which are the factors of our theoretical framework, are assimilated. The four-factor solution had the following factors: emotion, interest, attention, and hurdle, as expected from our theoretical framework. The lyrical appraisal factor, however, did not clearly emerge.

#### Reliability of the Music Receptivity Scale

##### Internal consistency

The overall internal consistency measured using Cronbach’s alpha was 0.89. For the 20-item four-factor solution, the alpha values were 0.81 (emotion), 0.84 (interest), 0.68 (attention), and 0.59 (hurdle). For the 20-item two-factor solution, the alpha values were 0.87 (affect) and 0.75 (attention).

##### Test–retest reliability

Test–retest was conducted on two different occasions, after 15 days and after 30 days. The test–retest reliability was found to be very high, *r*(45) = 0.87, *p* < 0.001 for the 15 day interval and *r*(49) = 0.91, *p* < 0.001 for a 30 day interval. This suggests high temporal stability of the tool. This high consistency can mean that both the stability of the construct and the consistency of the musical piece influence the respondents.

#### Validation of the First Item

The first item in the Music Receptivity Scale attempts to capture the type of emotion captured after listening to the given piece of music. The validity of this first item would differ from context to context. In our study, we played a musical piece that had predominantly positive emotion, especially sublime devotion, surrender, reflections, love, happiness, etc. In order to validate this, we used the principal component analysis to check if the responses get reduced to represent the main theme of the played song. The parallel analysis suggested three components. We observed that the first component had distinctively higher loadings in the pattern matrix compared with all the other components, and the first component’s eigenvalue was 9. The items of this component converged to the theme of sublime devotion, surrender, love, acceptance, etc., unambiguously capturing the theme of the played song. Hence, the first item also had good validity.

#### Factor Analysis of Musician Data (*n* = 44)

Following the previous analyses of the main study, two factors (affect and attention) were extracted, which reproduced the results of the main study. The mean MRS score of the musician group was significantly higher than that of the main study group, *t*(69.17) = 5.515, *p* < 0.001, *d* = 0.46. Also, the variance of the musician group was significantly lower than that of the main study group, *[F*(1, 355) = 4.89, *p* = 0.028]. This suggests a likely discriminant validity of the MRS tool in this analysis between musicians and the general population.

Considering the factor analysis results of the three subsets of data, we propose a two-factor solution for the Music Receptivity Scale to be used for all general purposes, and for clinical purposes, a four-factor solution would be recommended.

### Influence of Social Desirability

In any self-report measures, an element of social desirability may be present in the responses. Hence, to assess that, a social desirability scale was used. The correlation between the total music receptivity score and the total social desirability score was not statistically significant, *r*(310) = 0.01, *p* = 0.834, indicating that in this study sample, social desirability did not influence music receptivity and suggested that this construct is not socially sensitive. However, this particular result needs to be reproduced across study setups before it can be generalized.

## Discussion

We intended to develop an instrument to measure music receptivity, field test it, and assess its psychometric properties, and as a culmination, we developed a 20-item questionnaire having four domains (emotional experience, interest, attention, hurdles) and a shorter 12-item version of the same. Even though the five-factor model which we postulated in our conceptual model did not emerge, we suggest that some of the components are not psychometrically integrated, but conceptually integrated, and therefore, should be analyzed and separately interpreted. We excluded items 3 and 18 (items assessing hurdles) from the instrument and propose to add them as part of the set of instructions to ensure that confounders do not exist. We expected lyrical appraisal would come up as a separate domain of music receptivity, indicative of the report of Besson et al. (1998) saying listeners independently process lyrics and tunes. However, lyrical appraisal cannot be viewed as a watertight compartment as it dynamically interacts with emotional experience along with other factors and cumulatively contributes to the music receptivity score. Also looking at the wordings of the items measuring lyrical appraisal, two of the items emphasize feeling/emotional aspects, e.g., “did not like the lyrics” and “lyrics of the music moved me/touched my heart,” and hence, the domain of lyrical appraisal got submerged into the emotional experience domain. Lyrical appraisal did not come out as a standalone domain, partly due to the overlap of the two domains. However, we strongly propose that lyrical appraisal must form an independent domain, as it is important to measure it separately, especially in clinical settings where music is administered as therapy. The following study supports our contention where it was shown that happy music induced a higher degree of positive valance in “without lyrics” condition contrasted against “with lyrics” condition, and this study also clearly distinguished between experience of music with and without lyrics (Brattico et al., 2011). Therefore, it reinforces the idea that all the external and internal cues associated with a piece of music are precursors to activation of various mental representations, and once they occur, corresponding emotions are experienced.

The two-factor solution was a reduced item version, and it yielded the two most important metacomponents of music receptivity, i.e., affect and attention. The affect domain included interest and emotional experience, whereas attention remained as a separate factor. We also observed that musicians had significantly higher music receptivity scores compared with non-musicians. The musicians had lower variance in the music receptivity score compared with the non-musicians. These results may be considered as an initial evidence of discriminant validity of the MRS. However, more studies are needed to understand the different characteristics of musicianship on MRS scores. We, however, note that the information about the nature of musicianship in the general population study (*n* = 313) were not collected. There could have been some musicians in this group. The implication of this limitation is that it could have overestimated the MRS scores in the general population study and reduced the magnitude of difference in MRS scores between the musician and non-musician groups. As this is likely to have imparted a type II error, we consider our inference would still hold in future studies, when we control for musicianship in the general population. As far as the reproducibility of the results or the structure of the construct music receptivity is concerned, we expect it to be reproducible across different setups, as evident through our combined analysis of all the three sample sets yielding a similar factor structure.

The well-brought out domains are attention, interest, emotional experience, and hurdles, whereas lyrical appraisal merged into the domain of emotional experience. Considering the potential clinical applications of this tool, some of the items have been retained in the tool even though their removal would have given a high factor loading in factor analysis, for example, items 10 and 16 which are quite relevant in traumatic or clinical conditions. The first item of the Music Receptivity Scale appraises the nature and intensity of subjective feelings and emotions evoked in an individual while listening to a given piece of music. This is something similar to the Geneva Emotional Music Scales (GEMS), which has 45 items depicting various emotions that can be induced through music, and it has also been grouped into nine categories of emotion groups (Zentner et al., 2008). The first item of the Music Receptivity Scale has a similar structure; however, instead of using the GEMS, we used labels of commonly experienced emotions in music listening and also added a few other components like surrender, seeking mercy, etc. assuming that they would be more culturally relevant in an Indian context. The revised version of this scale, the Geneva Music-Induced Affect Checklist (GEMIAC), in which extra dimensions were added, had similar disparity. In this tool, the intensity and frequency of affective response are presented (Coutinho and Scherer, 2017).

The MRS has close resemblance with a few other tools. The Absorption in Music Scale was developed to identify people who might be especially responsive to music. It has domains like attention, altered sense of reality, access to old memories, increased imagery, duration of music listening, knowledge of music or artist, and belief mood affected by music. The working definition of absorption presented in this work, “Willingness to be drawn in deeply, without distraction, is called absorption” (Tellegen and Atkinson, 1974), has close resemblance with music receptivity. In the MRS, the interest and the emotion would define the willingness, and attention and hurdles would relate to external distractions (Sandstrom and Russo, 2013). The Absorption in Music Scale was also found to be assessing listening habits and preferences and the ability of music to influence one’s mood. Individual differences in absorption can predict differences in depth of emotional responses to music, which is also an aim of the Music Receptivity Scale. Absorption was found to be a significant indicator of post-stressor physiological recovery. We assume that further predictive validity studies using the MRS can also show similar results in clinical practices. In this reported study, the authors have used only one type of music, which has been highlighted as a limitation.

Intensity of music involvement was measured using the Music Involvement Scale. Some of the conceptual ideas incorporated into this tool include physical reaction, emotion, perception, cognition, and trance-like experiences. Similarly, the MRS also has emotion, attention, and lyrical appraisal domains. In the Music Involvement Scale, the proposed domains were subclassified into finer aspects based on content analysis. However, in the MRS, the domains are broadly labeled. Additionally, physical reactions and trance-like experiences were included, which are not present in the MRS (Nagy and Szabó, 2004).

The Profile of Music Perception Skills, PROMS, measures various perceptual components of music like pitch, timbre, and rhythm. This questionnaire can assess musical abilities of even non-musicians. While PROMS provides the ability to comprehend perceptual features of music, MRS measures certain cognitively evolved constructs based on basic perceptual processes after listening to a piece of music. Hence, PROMS can help to establish the convergent validity of the MRS. It can be assumed that if situational factors are held constant, higher musical perceptual ability is likely to be associated with music receptivity (Law and Zentner, 2012).

The Music USE (MUSE) questionnaire assesses the engagement styles of eight different background music and provides an overall qualitative and quantitative measure of music use. Four distinct engagement styles—cognitive and emotion regulation, engaged production, social connection, and dance and physical exercise—are presented (Chin and Rickard, 2012). The cognitive and emotion regulation domain particularly has some similarity to the MRS, where the emotion and interest domains are related. However, the items in this questionnaire are not contextually mapped to the given piece of music. We consider that, at the time of listening to a musical piece, emotion regulation ability is less important than other automatic psychological processes.

The Adaptive Functions of Music Listening Scale is another scale that measures various music listening functions related to general well-being. Among the various affective, social, and cognitive functions derived from music listening, strong emotional experiences and cognitive regulation appear similar to the MRS domains of emotion and attention (Groarke and Hogan, 2018).

As a general remark, we can say that for all these constructs, if we look into the trait part, the higher scores of the MRS are more likely to be associated with the higher scores in these traits, given that the situational factors are not unduly influenced. It can also be observed that most of these closely related tools attempt to measure some trait aspects in an individual, and these tools can be administered most often without any musical piece presented to the participants. However, in music therapy setups, it is essential to evaluate the mental status of an individual associated with situational factors and trait aspects together. These two aspects together would define to what extent an individual can have receptivity to the given piece of music. In music, where there is a constant interaction between the person and the music, distinguishing or labeling a construct as trait or state sometimes becomes challenging as both trait and state characteristics might be present.

The concept of music receptivity may have far-reaching implications in relation to the various existing theories in music psychology, education, research, and clinical practices. The Music Receptivity Scale may indicate intrinsic and extrinsic motivation. It may also possibly predict musical engagement. To further evaluate the predictive validity of the Music Receptivity Scale, we could possibly study the personality dimensions associated with music receptivity. A study investigating who should study music found a correlation with musical outcome and Holland’s personality concept of vocational personality and environments (Cevik et al., 2013). Another study based on the Savanna–IQ interaction hypothesis reported that intelligence is related with preference for instrumental music over vocal music and also associated with reflective, intense, and sophisticated types of music, which gives an insight that higher-order appraisal requires higher cognitive functions (Račevska and Tadinac, 2019). Musical training is another important factor that would be a strong predictor of music receptivity. It has been reported that musical training is associated with perceptual and cognitive skills, including executive functions and general intelligence (Criscuolo et al., 2019). We can anticipate higher musical training or musical inclination to be associated with higher music receptivity. Specifically, the attention, interest, and emotion domains of the MRS will be closely related to musical training.

Modulation and appraisal of emotions while listening to music and the way different experiences are felt have been an important focus of many studies. Prior attempts were made to develop scales to measure attitude toward music (Solomon and Edwards, 1971). Also, other similar constructs like the Musical Sophistication Index and the Brief Music in Mood Regulation Scale (B-MMR) emphasize the importance of emotions. The Musical Sophistication Index measures musical skills, abilities, and behavior, such as active engagement, perceptual abilities, musical training, singing abilities, and emotion (Degrave and Dedonder, 2019). The B-MMR attempts to measure seven different music-related mood regulation strategies (Saarikallio, 2008).

The trajectory from music listening to behavioral responses can be staged into three phases—music listening (interactive phase), music processing (appraisal phase), and response exhibition (response phase). In the first stage of the interactive phase, the perceptual abilities would play a major role in deciding the music receptivity. In the interactive phase, feature extraction (timbre, pitch), Gestalt formation, auditory sensory memory, analysis of intervals, structure building, structural reanalysis and repair, vitalization, and premotor action might occur. This description, according to the neurocognitive model of music perception (Koelsch, 2011), describes the whole spectrum of events involved in the process. These neurocognitive music perception events can modulate music receptivity in various ways. To start with, initial attention would be enhanced if the given piece of music contains relevant acoustic information that would help form Gestalten consolidation, triggering corresponding auditory sensory memories that would further activate the interest. If these components happen without much barrier, then it would lead to higher music receptivity. As an outcome of that, higher-order mind–body responses can be seen at the physical level (as chills, etc.) and mental level (as esthetic awe). Hence, the initial exposure to a music piece has an important function to trigger relevant acoustic information that matches with a person’s music identity. Here, we would like to emphasize that the initial perceptual abilities can be influenced by situational factors like the quality of music, listening ambiance, momentary state of mind, and mood. These are closely related to the hurdle dimension of the Music Receptivity Scale.

Experiencing higher-order mind–body experiences such as physiological chills and thrills, feeling moved, and esthetic awe is a complex phenomenon (Konečni, 2011). There are many perspectives presented in the scientific literature. In a study, participants listened to their preferred choice of music, and later, they were assessed using the Tellegen Absorption Scale and Phenomenology of Consciousness Inventory. They showed two types of deep absorptions—zoning-in and tuning-in—and showed the interacting role of cognitive and affective systems (Vroegh, 2019). Similarly, the embodied cognition in music was suggested to have two levels: the surface level where bodily movements get activated through psychomotor movements and the deep level of embodied cognition that integrates other perceptual properties of music and synergistically paves the path to experience various higher-order musical experiences. This feature was hinted as an important factor in distinguishing different levels of musicianship and their brain plasticity (Korsakova-Kreyn, 2018). This intricate phenomenon can be understood using the spreading activation theory. According to this theory, deeper experiences in music listening can be brought about by forming relevant mental representations and suitably activating it at a later time. These mental representations form the musical identity of a person. These mental representations can be formed through active engagement with music and also by mere exposure, perhaps through subliminal pathways as in the case with passive listening. If some of these mental representations are activated through the spreading mechanism, then higher-order mind–body experiences may be induced. External environmental factors are also very important in the process of initiating suitable mental representations. The spreading activation theory explains many of the complex interrelationships between music listening, experiencing higher-order emotions, and social interactions (Schubert et al., 2014). The implication of this is that by carefully modulating these factors, music receptivity could be possibly regulated.

Studying music receptivity further may help us understand the theories related to the experience of higher-order mind–body phenomena in music psychology. For further ecological validation, we would need to carefully design and control the experimental conditions to have experience of such higher-order emotions and then study using this tool. There can be many other confounders; for instance, in the study, it was shown that felt emotions and perceived emotions may be quite different. Sad music sometimes appears pleasant; owing to that, though sad music was perceived as sad, the actual experience felt by the participants was pleasant, and this strongly emphasizes that the mental representation through which a person feels ultimately is very important (Kawakami et al., 2013). A similar idea is also echoed in another article where the authors suggest a constructionist perspective of emotion induction through music listening. They argue that music does not essentially induce basic emotions, rather through modulation of core affect (valence and arousal), appropriate mental representations are activated and bring out a spectrum of musical emotions (Cespedes-Guevara and Eerola, 2018). It was also suggested that music listening may bring about mood modulation. Even though these moods may be variable and subjective, they can be linked to a specific emotion, as music listening deliberately aligns feelings to a particular set of emotions. This implies a cumulative effect leading to experiencing higher-order mind–body experiences (Goffin, 2014).

Hence, it can be seen that the construct music receptivity has wide linkages with many other theories in music and further studies using this tool can give much insightful information in the future.

### Limitations and Future Scope of the Study

We believe the Music Receptivity Scale can be a potential tool for clinical applications. However, in this study, we could not validate the instrument using a clinical sample. As clinical setups are varied, we require multiple studies designed to suit specific clinical conditions. Music receptivity is a generalized concept that can be applied to varied contexts in music such as music education, music in daily life, music for well-being, and so on. Another limitation of our study was that we used only one musical stimulus, and with that, it would be difficult to generalize MRS’s performance across different themes of music. As the music receptivity scores would depend on different situational factors, the state aspect of the MRS would require further investigation. Therefore, we would need studies in all these contexts to evaluate the overall ecological validity of the tool. Similarly, music receptivity may be influenced by age, socioeconomic strata, etc., which we could not address in our study. We also could not investigate other approaches to establish construct validity except factor analysis. Looking at convergent and discriminant validity together to establish construct validity would have given an additional dimension to the construct validity. Being in the initial stage of development, where our focus was mainly to identify and ascertain the most important domains of music receptivity (also which can be measured and manipulated in future experimental studies—which can stand future theory testing), we followed the factor analysis approach. Furthermore, correlational-based convergent and discriminant approaches are considered weaker, especially in the very initial stage of the tool development (Strauss and Smith, 2009). Hence, this should be taken up in future independent studies where the focus should be to investigate the interaction of the MRS with other existing constructs. Using exploratory factor analysis has a number of limitations, and the outcome of this study can only be considered as an initial evidence for the Music Receptivity Scale. In future studies, more robust analysis using the confirmatory factor analysis framework can be adopted (Carlucci et al., 2015; Saggino et al., 2017). Future research studies demonstrating validity of music receptivity across these strata would add to the overall strength of the tool. Apart from that, we observed as a general condition that the ambiance of conducting the assessment is very important and that has to be carefully controlled for optimal results. Lastly, lyrical appraisal did not emerge as a strong and independent domain, though we expected that based on our theoretical framework. Future studies can attempt on experiments “with” and “without” lyrics.

In this work, we were just able to present MRS as a useful tool for daily practice in music therapy setups. The focus is easy administration, scoring, and quick evaluation of the receptivity of a person to the given piece of music, at that given time point. The domains of the MRS are more likely to be associated with relevant subdomains of different scales as mentioned earlier in the Discussion section. However, such emerging relationship is limited to the consistency of the situational criteria. Hence, such comparison studies should be carefully conducted by suitably controlling for various situational factors. This would pose a methodological challenge in future concurrent validity studies.

In a clinical setting, the Music Receptivity Scale would enable the music therapist to continually evaluate client responses to standardized music interventions and help them manage client databases. This would facilitate the therapist to administer customized music interventions to an individual or a select group of individuals, over a longer course of time. One of the scopes of this study was to develop a feedback mechanism that could assist the music therapist in a clinical music therapy setting to identify unique patient characteristics in music therapy. Furthermore, this psychometric assessment could be integrally employed as a module in a smartphone-based application, which could enable automating music therapy in clinical settings. Automated music therapy in clinical settings would largely reduce the effort and frequency of intervention from the music therapist, henceforth bringing down the overall cost incurred by the clients undertaking music therapy.

To conclude, a new construct of music receptivity was defined, and its psychometric validation was proposed. Overall, the tool had 20 items in the long form and 12 items in the short version. Two solutions were observed for the factor structure: one with a two-factor structure of affect and attention and another solution of four factors, where affect diverged into interest and emotions, whereas attention and hurdle remained as next emerging factors. Implication of music receptivity with other existing theories in the field of music has also been discussed.

## Data Availability Statement

The datasets presented in this study can be found in online repositories. The names of the repository/repositories and accession number(s) can be found below: Open Science Framework link: https://osf.io/v8jb9/.

## Ethics Statement

The studies involving human participants were reviewed and approved by the IEC of Swami Vivekananda Yoga Anusandhana Samsthana, Bengaluru, India. The patients/participants provided their written informed consent to participate in this study.

## Author Contributions

MG contributed toward the conceptualization, literature review, data collection, analysis, and manuscript writing. JI contributed toward the conceptualization, literature review, analysis, manuscript writing, and editing. Both authors contributed to the article and approved the submitted version.

## Conflict of Interest

The authors declare that the research was conducted in the absence of any commercial or financial relationships that could be construed as a potential conflict of interest.
